# Supplementary material for: Influence of InP/ZnS Quantum Dots on Thermodynamic Properties and Morphology of the DPPC/DPPG Monolayers at Different Temperatures
Source: Molecules. 2023 Jan 22;28(3):1118. doi: 10.3390/molecules28031118 (PMC9920855; doi:10.3390/molecules28031118)
Supplement: Supplementary file 1 [file molecules-28-01118-s001.zip › molecules-2167229-supplementary.pdf]

supplementary data

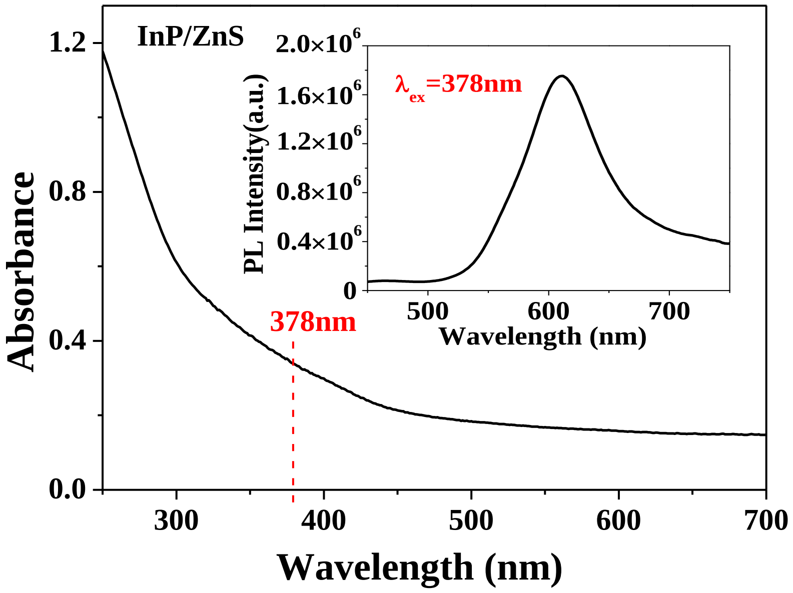

Figure S1 Absorption spectra and photoluminescence spectra of InP/ZnS QDs without surface groups.

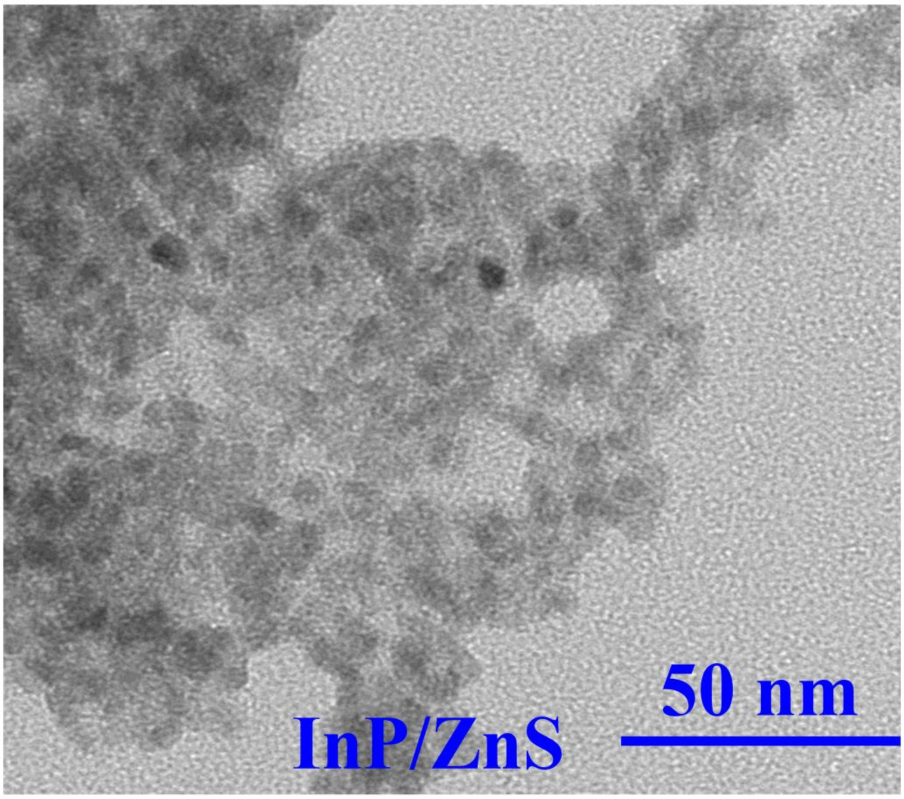

Figure S2 The TEM images of InP/ZnS QDs without surface groups.
